# Pathological mechanisms of inflammatory responses in atherosclerosis and the multi-target regulatory effects of traditional Chinese medicine

**DOI:** 10.3389/fcvm.2026.1843775

**Published:** 2026-07-24

**Authors:** Le Zhang, Zihan Chang, Aiying Li, Haijun Wang, Yu Liu

**Affiliations:** 1 Department of Biochemistry and Molecular Biology, College of Pharmacy, Hebei University of Chinese Medicine, Shijiazhuang, China; 2 College of Pharmacy, Hebei University of Chinese Medicine, Hebei Key Laboratory of Chinese Medicine Research on Cardio-cerebrovascular Disease, Shijiazhuang, Hebei, China; 3 College of Pharmacy, Hebei University of Chinese Medicine, Hebei Higher Education Institute Applied Technology Research Center on TCM Development and Industrialization, Shijiazhuang, Hebei, China; 4 Department of Surgery, Second Affiliated Hospital of Hebei Medical University, Shijiazhuang, Hebei, China

**Keywords:** atherosclerosis, inflammatory response, oxidative stress, signaling pathways, traditional Chinese medicine

## Abstract

Atherosclerosis (AS) is a vascular disease characterized by chronic inflammation and represents a major pathological basis for cardiovascular events such as myocardial infarction and ischemic stroke. During disease initiation and progression, endothelial dysfunction, lipid retention, sustained recruitment of immune cells, and aberrant activation of inflammatory signaling pathways interact to establish a self-perpetuating inflammatory microenvironment. This process is accompanied by dysregulation of inflammatory cytokine networks, persistent activation of key signaling pathways such as nuclear factor-*κ*B (NF-*κ*B) and mitogen-activated protein kinase (MAPK), increased oxidative stress, and enhanced thrombotic susceptibility, ultimately driving plaque formation, destabilization, and acute cardiovascular events. Given that these pathological mechanisms involve multiple cell types, interconnected signaling axes, and dynamic changes across different disease stages, single-target therapeutic strategies often show limited efficacy in long-term disease control. Traditional Chinese medicine (TCM), characterized by its multi-component, multi-target, and system-level regulatory properties, has attracted increasing attention for its potential to modulate inflammation-related pathological processes in atherosclerosis at multiple levels. Accumulating evidence indicates that TCM and its bioactive constituents can regulate inflammatory cytokine expression, inhibit NF-*κ*B- and MAPK-mediated inflammatory signaling, alleviate oxidative stress, improve endothelial function, and reduce thrombotic susceptibility, thereby collectively attenuating chronic inflammatory burden and promoting plaque stability. This review focuses on inflammation-associated key pathological processes in atherosclerosis and systematically summarizes recent advances in the regulatory effects of TCM on inflammatory responses, signaling pathways, oxidative stress, and thrombosis. The aim is to provide mechanistic insights that may support integrated strategies for atherosclerosis prevention and treatment, as well as the modernization of TCM research.

## 1 Introduction

Atherosclerosis (AS) is a common pathological basis of multiple cardiovascular diseases and is closely associated with coronary atherosclerotic heart disease, ischemic stroke, and peripheral arterial disease (1). According to data from the World Health Organization, cardiovascular diseases remain the leading cause of death worldwide, accounting for approximately 44% of all deaths from non-communicable diseases and resulting in about 17.9 million deaths annually, a substantial proportion of which are closely related to atherosclerosis (2). In addition to its severe health burden, atherosclerosis-related diseases exert persistent and profound impacts on socioeconomic systems. Cardiovascular events caused by atherosclerosis are often accompanied by long-term treatment, repeated hospitalizations, and functional impairment, placing significant pressure on healthcare resource allocation and social productivity. With the continuous rise in medical costs, health expenditures associated with atherosclerosis-related diseases have become a major challenge for global public health systems, particularly in low- and middle-income countries (3).

**Figure 1:**
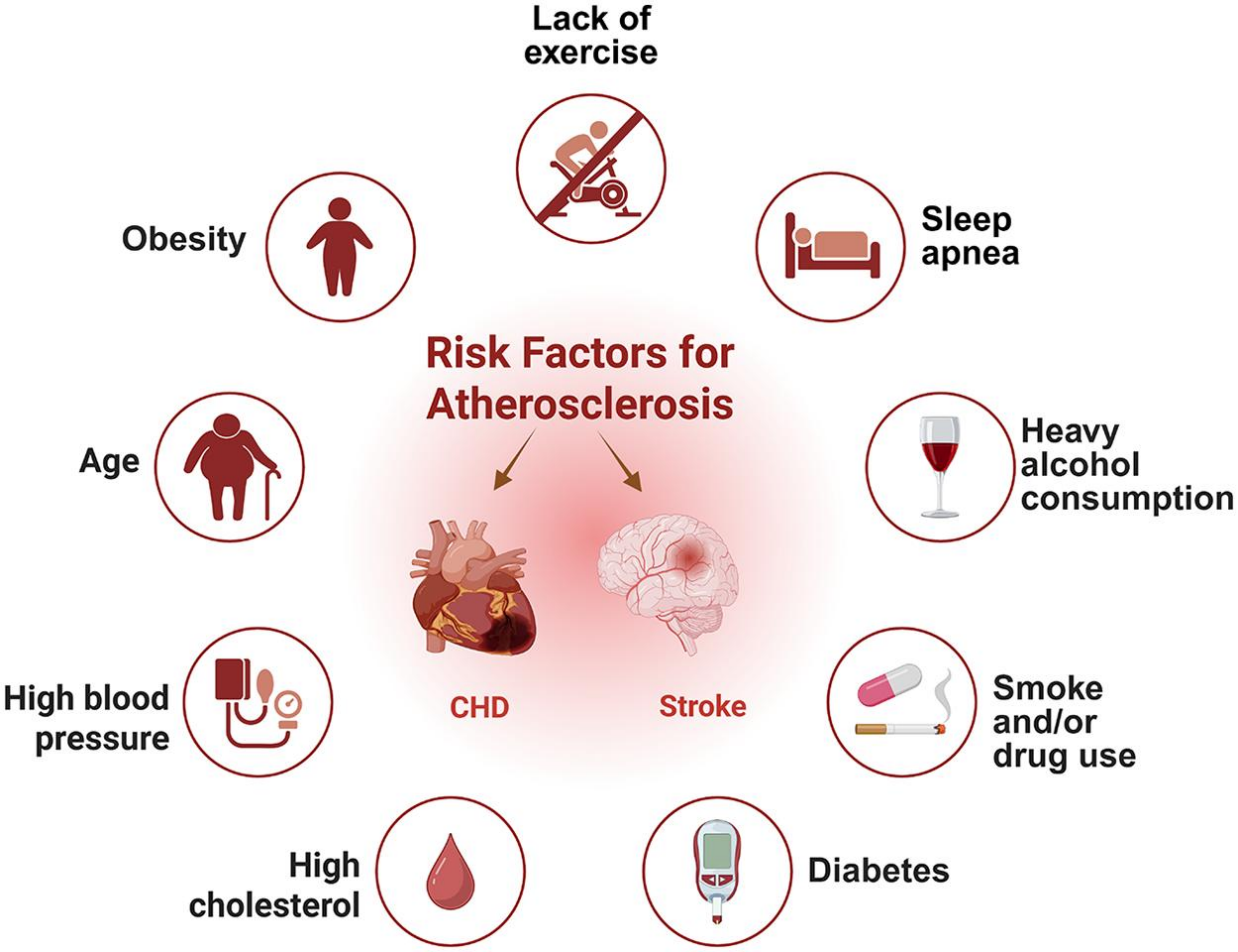
Risk factors for atherosclerosis this infographic illustrates various risk factors for atherosclerosis, including lack of exercise, obesity, age, smoking and/or drug use, high blood pressure, high cholesterol, diabetes, heavy alcohol consumption, and sleep apnea. These factors are closely associated with the risks of Coronary Heart Disease (CHD) and Stroke.

The development of atherosclerosis is closely associated with multiple cardiovascular risk factors, including aging, hypertension, hyperlipidemia, diabetes mellitus, smoking, unhealthy dietary habits, and bacterial infections (Figure 1). These factors impair endothelial function through different pathways, creating conditions favorable for lipid retention, inflammatory activation, and plaque formation (4). Although therapeutic strategies primarily targeting lipid metabolism regulation and hemodynamic improvement have achieved certain success in reducing cardiovascular event risk, the initiation and progression of atherosclerosis cannot be fully explained by lipid deposition or vascular stenosis alone. Increasing evidence has highlighted the central role of inflammation in the initiation and progression of atherosclerosis, supporting the concept that atherosclerosis is a vascular disease driven predominantly by chronic inflammatory responses (5).

The early stage of atherosclerosis is typically triggered by endothelial injury. Following endothelial damage, low-density lipoprotein (LDL) oxidation and its metabolic products accumulate within the vascular wall and serve as major initiators of local inflammatory responses (6). oxidized low-density lipoprotein (oxLDL) not only promotes immune cell infiltration but also induces the release of inflammatory mediators, further activating endothelial cells and other immune cells and sustaining local inflammatory states (7). With the continuous accumulation of inflammatory mediators, extracellular matrix remodeling occurs within the vascular wall, and vascular smooth muscle cells proliferate and migrate into the intima, contributing to plaque formation and fibrous cap development while influencing plaque stability (8). As inflammation becomes chronic, sustained immune cell activation enhances proteolytic activity and extracellular matrix degradation, shifting plaques from a stable to a vulnerable phenotype (9). Persistent activation of local immune cells promotes plaque enlargement and destabilization; rupture of the fibrous cap exposes plaque contents to the bloodstream, triggering thrombosis and increasing the risk of acute cardiovascular events (9, 10). Taken together, inflammation not only accelerates atherosclerotic lesion formation but also critically determines plaque stability, making inflammation control a key therapeutic target in atherosclerosis (11).

Given that inflammatory responses in atherosclerosis involve multiple cell types, diverse signaling axes, and dynamic changes across different disease stages, single-target intervention strategies often exhibit limitations in long-term efficacy and system-level regulation (12). Against this background, TCM, characterized by its multi-component, multi-target, and holistic regulatory properties, has gained increasing attention in the intervention of chronic inflammatory diseases (13). In recent years, accumulating studies have elucidated the roles of bioactive components derived from TCM at molecular and signaling network levels, demonstrating their regulatory effects on inflammation, oxidative stress suppression, endothelial protection, and modulation of thrombotic susceptibility (14, 15). However, these findings remain relatively fragmented, and systematic integration and mechanistic synthesis centered on inflammatory pathological networks are still lacking.

Based on this background, the present review systematically summarizes recent advances in the pathogenesis of atherosclerosis, with a particular focus on inflammation-related key pathological processes during disease initiation and progression. Furthermore, it synthesizes current evidence regarding the mechanisms by which TCM and its bioactive components regulate inflammatory mediators, intervene in signaling pathways, modulate oxidative stress, and exert anti-thrombotic effects. This review aims to provide mechanistic insights into the potential value of TCM in inflammation-centered regulation of atherosclerosis and to offer references for future basic research and integrated prevention and treatment strategies.

## 2 Research progress in atherosclerosis

**Figure 2:**
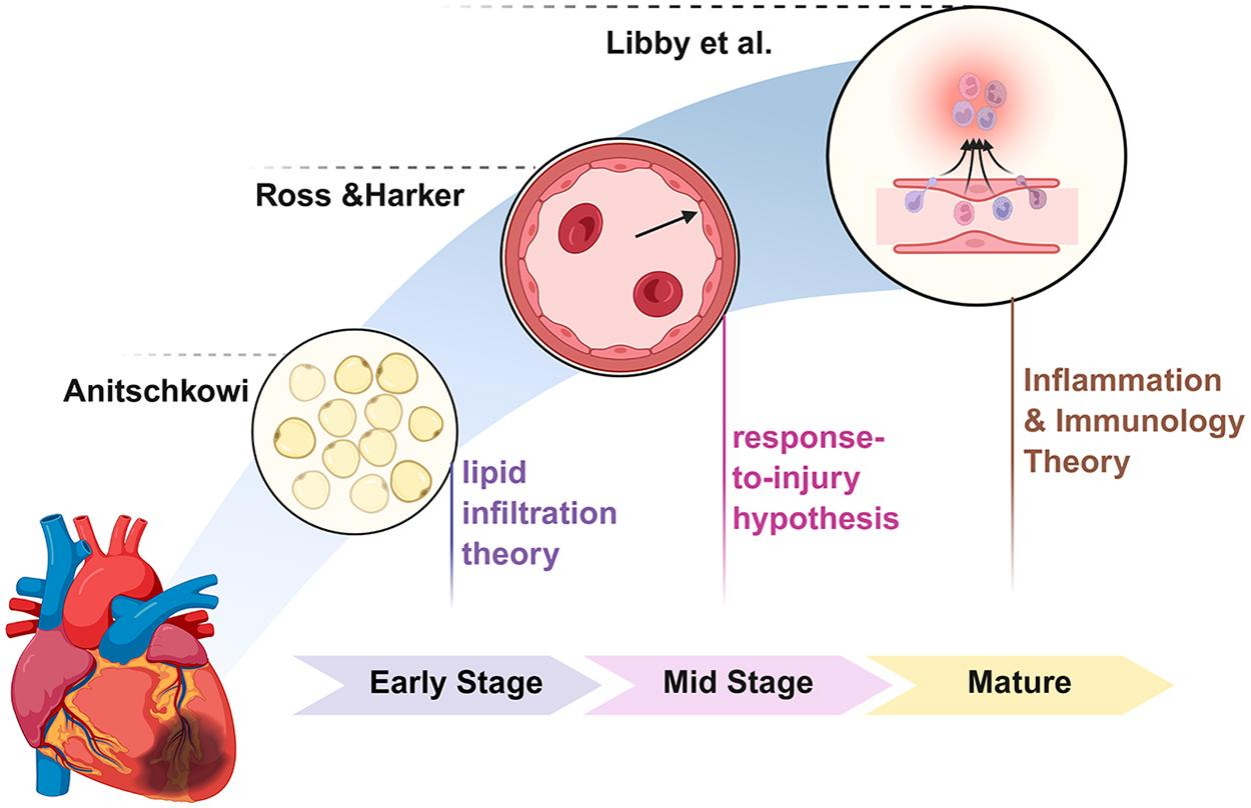
Evolution of theories in atherosclerosis research this infographic illustrates the evolution of theories in atherosclerosis research. It progresses from the early lipid infiltration theory (Anitschkow) to the response-to-injury hypothesis (Ross & Harker), and finally to the Inflammation & Immunology Theory proposed by Libby et al., representing the deepening understanding and development of the mechanisms behind atherosclerosis.

To better understand the central role of inflammation in atherosclerosis, it is necessary to review the historical evolution of concepts regarding the pathogenesis of this disease (Figure 2).

### 2.1 Lipid infiltration theory: early concepts centered on cholesterol deposition

The understanding of the pathogenesis of atherosclerosis has undergone continuous evolution. In the early 20th century, the Russian pathologist Anitschkow demonstrated, using a cholesterol-fed rabbit model, that cholesterol could accumulate within the arterial wall and give rise to typical atherosclerotic lesions. Based on these observations, he proposed the *lipid infiltration theory*, which suggested that elevated plasma cholesterol levels and its deposition within the vascular wall play a key role in the development of atherosclerosis (16). This theory dominated the field for an extended period and provided a theoretical basis for the association between hypercholesterolemia and atherosclerotic disease.

### 2.2 Response-to-injury hypothesis: proposal of active vascular wall responses

With advances in research, it became increasingly evident that lipid deposition alone could not fully explain the complex pathological processes underlying atherosclerosis. In the 1970s, Ross and Harker proposed the *response-to-injury hypothesis* based on systematic analyses of animal experiments and pathological studies, arguing that endothelial dysfunction or injury represents a critical initiating event in atherogenesis. Endothelial injury increases vascular permeability, facilitating lipid entry into the arterial wall, while simultaneously inducing platelet adhesion, monocyte recruitment, and vascular smooth muscle cell migration and proliferation, thereby promoting plaque formation and progression (17). This hypothesis linked lipid abnormalities with active cellular responses of the vascular wall and provided a more dynamic perspective on the mechanisms of atherosclerosis.

### 2.3 Inflammatory and immunological theory: atherosclerosis as a chronic inflammatory disease

From the late 20th to the early 21st century, advances in molecular biology and immunology further expanded perspectives on atherosclerosis research. Steinberg systematically reviewed the development of the cholesterol hypothesis and related clinical evidence, emphasizing that the retention and modification of low-density lipoprotein cholesterol within the arterial wall play a fundamental role in lesion formation. Large-scale clinical trials further confirmed that lowering blood cholesterol levels significantly reduces cardiovascular events and mortality, thereby establishing the pathogenic role of cholesterol in atherosclerosis (18).

From inflammatory and immunological perspectives, Libby and colleagues integrated previous findings and proposed that atherosclerosis should be regarded as a chronic vascular disease driven by sustained inflammatory and immune responses, occurring on the basis of LDL retention within the arterial wall and initiated by endothelial dysfunction. Inflammatory processes permeate all stages of disease development, progression, and complication formation, playing critical roles in plaque formation, regulation of plaque stability, and eventual plaque rupture (19–21).

In summary, the conceptual framework of atherosclerosis has evolved from early lipid-centered theories to an integrated model initiated by endothelial injury and sustained by inflammatory and immune responses. It is now widely recognized that atherosclerosis is a complex pathological process involving interactions among multiple cell types, signaling pathways, and disease stages, in which inflammation serves as a key link connecting lipid abnormalities, vascular injury, and clinical cardiovascular events.

## 3 Key inflammatory pathological processes in atherosclerosis

**Figure 3:**
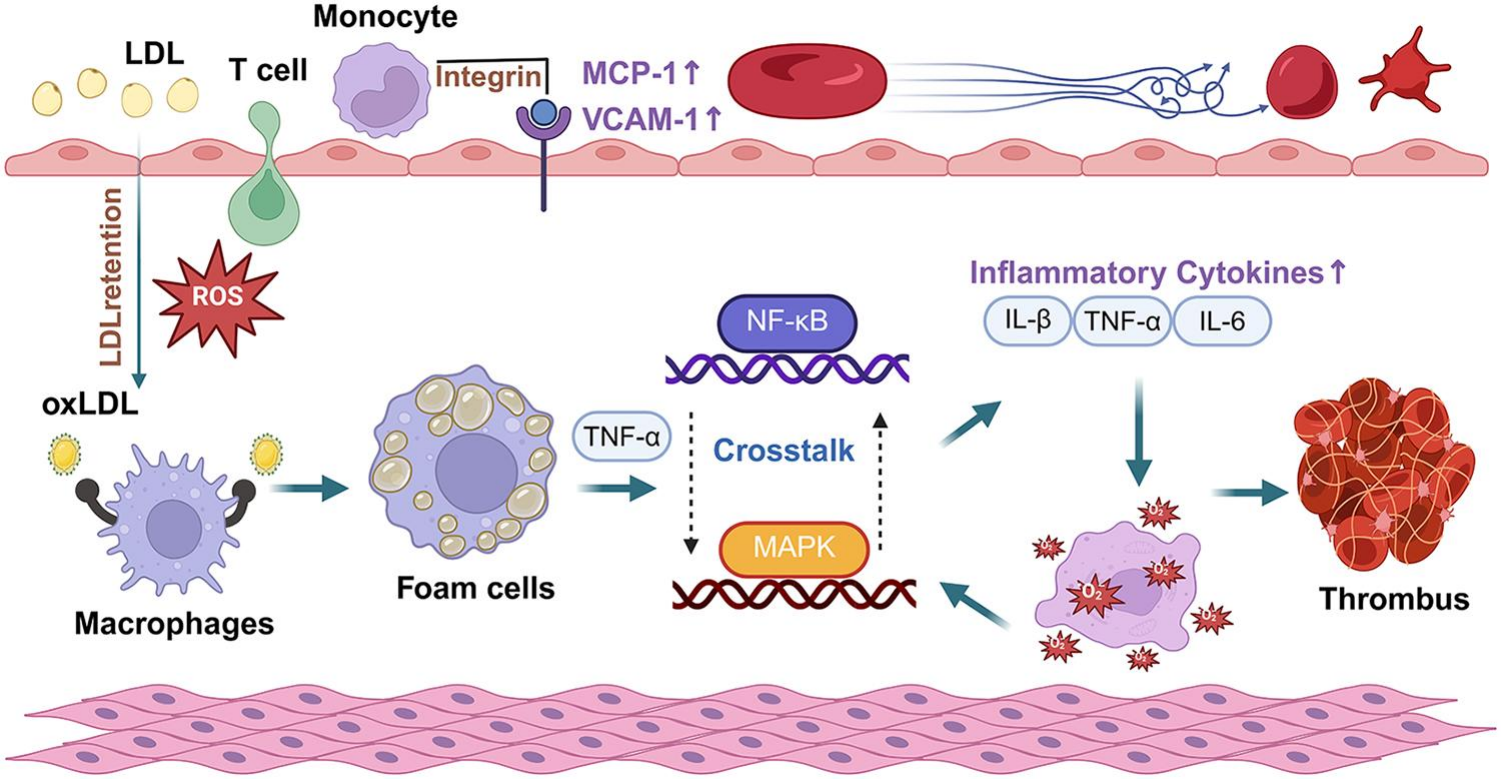
Mechanism of atherosclerosis this infographic illustrates the pathogenesis of atherosclerosis. First, LDL enters the vascular wall following endothelial injury, triggering an immune response. The expression of VCAM-1 and MCP-1 on endothelial cells is upregulated, promoting the adhesion and infiltration of monocytes and T cells, leading to foam cell formation. Oxidized LDL (oxLDL) further activates macrophages, transforming them into foam cells and increasing local inflammation. The release of inflammatory cytokines (such as TNF-α, IL-1β, and IL-6) amplifies the inflammatory response through the NF-*κ*B and MAPK pathways, promoting plaque formation and instability. Oxidative stress and inflammation interact with each other, amplifying the effects and contributing to thrombus formation, thus accelerating the progression of atherosclerosis.

AS is a chronic inflammatory disease involving complex interactions among metabolic disturbance, endothelial dysfunction, immune activation, and thrombotic responses. The key inflammatory pathological processes underlying disease initiation, progression, and plaque destabilization are illustrated in Figure 3.

### 3.1 Endothelial injury and initiation of inflammatory responses

Vascular endothelial cells play a crucial role in maintaining vascular homeostasis, regulating vascular permeability, and suppressing inflammatory responses. Endothelial dysfunction is widely recognized as an early event in the initiation and progression of atherosclerosis. Although various cardiovascular risk factors act through distinct mechanisms, their common consequence is the disruption of endothelial structure and function, leading to endothelial dysfunction, breakdown of vascular wall homeostasis, and subsequent initiation of local inflammatory responses. Previous studies have clearly demonstrated that endothelial injury represents the starting point of inflammatory responses in atherosclerosis and persists throughout the entire course of disease development (19, 22).

In addition to traditional cardiovascular risk factors, hemodynamic forces have emerged as important regulators of endothelial function during atherosclerosis development. Among these factors, wall shear stress (WSS), the tangential force generated by blood flow on the endothelial surface, plays a critical role in vascular homeostasis and plaque evolution. Disturbed flow patterns and low WSS can induce endothelial dysfunction through mechanotransduction-mediated signaling pathways, thereby promoting inflammatory gene expression, oxidative stress, and leukocyte adhesion, ultimately creating a pro-atherogenic vascular microenvironment. In contrast, increasing evidence suggests that regions exposed to high WSS are more closely associated with plaque destabilization, erosion, rupture, and acute coronary syndromes. These findings indicate that vascular fluid dynamics not only contribute to the initiation of atherosclerosis but also influence plaque progression and vulnerability throughout the disease course (23).

Following endothelial dysfunction, vascular permeability increases, allowing LDL to more readily accumulate within the vascular intima. Under conditions of local oxidative stress, retained LDL undergoes oxidative modification to form oxLDL. Accumulating evidence indicates that oxLDL exhibits potent pro-inflammatory activity by activating endothelial cells and promoting their transition toward a pro-inflammatory phenotype, thereby upregulating the expression of vascular cell adhesion molecule-1 (VCAM-1) and intercellular adhesion molecule-1 (ICAM-1) (24, 25).

The upregulation of adhesion molecules enhances interactions between inflammatory cells and endothelial cells, facilitating the adhesion of circulating monocytes and lymphocytes to the endothelial surface and their subsequent migration into the vascular intima. Meanwhile, activated endothelial cells continuously secrete inflammatory mediators, including monocyte chemoattractant protein-1 (MCP-1) and tumor necrosis factor-α (TNF-α), further promoting inflammatory cell recruitment and amplifying local inflammatory responses. The chemokine–receptor axis plays a critical role in this process, among which the MCP-1/CCR2 signaling pathway is particularly representative. Genetic or functional disruption of this pathway markedly attenuates atherosclerotic lesion development, highlighting inflammatory cell recruitment as a key event in atherogenesis (26).

### 3.2 Immune cell infiltration and foam cell formation

On the basis of endothelial dysfunction and local inflammatory responses, the recruitment and infiltration of immune cells into the vascular wall represent critical events in the development of atherosclerosis. Under inflammatory stimulation, injured endothelial cells upregulate various adhesion molecules and release chemotactic signals, thereby promoting the adhesion, rolling, and transendothelial migration of circulating immune cells into the vascular intima, where they participate in and sustain local inflammatory responses. Among these immune cells, monocytes are considered the earliest and most predominant contributors to this process, and their continuous recruitment provides a cellular foundation for subsequent lesion progression (27, 28).

After entering the vascular intima, monocytes differentiate into macrophages under the influence of local cytokines and growth factors. Macrophages continuously internalize oxidized or other modified forms of low-density lipoprotein through scavenger receptor–mediated pathways, leading to progressive intracellular lipid accumulation and eventual formation of foam cells. The emergence of foam cells is a hallmark of early atherosclerotic lesions and reflects the cellular-level interplay between dysregulated lipid metabolism and inflammatory responses (21, 29).

In addition to the monocyte–macrophage system, the immunoregulatory roles of T lymphocytes within the plaque microenvironment have received increasing attention. Distinct T-cell subsets can modulate macrophage activation status, lipid-handling capacity, and the intensity of local inflammatory responses through cytokine secretion, thereby influencing foam cell formation and immune homeostasis within atherosclerotic plaques (30).

Accumulating evidence further supports the involvement of adaptive immune responses in atherosclerosis. Activated CD4^+^ T cells can amplify local inflammatory responses through cytokine secretion and immune regulation within atherosclerotic lesions. Moreover, dysregulated T-cell activation has been associated with plaque instability and acute coronary syndromes. Flego et al. demonstrated that helper T cells from patients with acute coronary syndrome exhibited reduced CD31 expression and impaired CD31-mediated inhibitory signaling, resulting in enhanced T-cell activation and altered MAPK signaling. These findings suggest that abnormalities in T-cell immune regulation may contribute to plaque progression and destabilization (31).

Foam cells are not merely passive lipid storage cells but also represent important inflammatory effector cells within the plaque microenvironment. They can secrete a variety of inflammatory mediators, chemokines, and reactive oxygen species, thereby further amplifying local inflammatory responses. As the disease progresses, apoptosis or necrosis of foam cells increases. When the clearance of apoptotic cells is impaired, cellular debris and lipids gradually accumulate within the plaque and form a necrotic core, ultimately driving plaque evolution toward increased complexity and instability (32).

### 3.3 Inflammatory mediators driving plaque formation and destabilization

Following sustained immune cell infiltration and foam cell formation, various inflammatory mediators—including pro- and anti-inflammatory cytokines, chemokines, and other immunoregulatory molecules—gradually emerge as key signaling factors driving plaque formation and progression. During the early stages of atherosclerotic lesion development, endothelial dysfunction and lipid deposition induce the expression and release of multiple pro-inflammatory cytokines and chemokines, thereby shaping a local inflammatory microenvironment conducive to disease progression. Representative inflammatory mediators such as TNF-α, interleukin-1β (IL-1β), interleukin-6 (IL-6), and MCP-1 enhance immune cell recruitment, retention, and activation, promote monocyte differentiation into macrophages, and accelerate foam cell formation, collectively driving the initiation and expansion of atherosclerotic plaques (33, 34). In addition, these inflammatory mediators can directly act on vascular smooth muscle cells, regulating their migration, proliferation, and phenotypic switching, and thereby participating in early structural remodeling of the vascular wall (35). A summary of the major inflammatory mediators and regulatory molecules involved in atherosclerosis and their principal biological functions is presented in Table 1.

**Table 1:** Major inflammatory mediators and regulatory molecules involved in the pathogenesis of atherosclerosis.

Functional Category	Molecule	Major Functions in Atherosclerosis	References
Pro-inflammatory cytokine	TNF-α	Activates endothelial cells and macrophages, increases vascular permeability, promotes immune cell infiltration, amplifies inflammatory responses, and enhances plaque instability.	(33)
	IL-1β	Induces inflammatory activation of endothelial and smooth muscle cells, promotes adhesion molecule expression, and accelerates foam cell formation and plaque progression.	(33)
	IL-6	Mediates acute-phase responses, enhances immune cell activation, promotes vascular remodeling, and contributes to plaque progression and destabilization.	(33)
Chemokine	MCP-1 (CCL2)	Recruits circulating monocytes into the vascular wall, promotes macrophage accumulation and foam cell formation, and sustains local inflammation.	(34)
Anti-inflammatory cytokine	IL-10	Suppresses pro-inflammatory cytokine production, limits macrophage activation, promotes inflammation resolution, and stabilizes plaques.	(37)
	TGF-β	Promotes extracellular matrix deposition and fibrous cap formation while suppressing excessive immune activation.	(37)
Endothelial function regulator	eNOS	Catalyzes nitric oxide production, maintains endothelial homeostasis, inhibits leukocyte adhesion and platelet aggregation.	(85)
	NO	Preserves vasodilation, suppresses endothelial activation, inhibits vascular smooth muscle proliferation, and counteracts inflammation and thrombosis.	(85)
Adhesion molecule	VCAM-1	Mediates monocyte and lymphocyte adhesion to activated endothelial cells, facilitating inflammatory cell recruitment.	(86, 87)
	ICAM-1	Promotes firm adhesion and transendothelial migration of leukocytes into vascular lesions.	(87)
Pattern recognition receptor	TLR4	Recognizes oxLDL and endogenous danger signals, activates innate immune responses, and amplifies inflammatory signaling.	(88)
	NLRP3	Senses cellular stress signals, assembles the inflammasome, activates caspase-1, and promotes IL-1β/IL-18 maturation and pyroptosis.	(89)
Oxidative stress regulator	ROS	Induces LDL oxidation and endothelial dysfunction, activates inflammatory signaling pathways, and accelerates plaque progression.	(90)
Matrix remodeling enzyme	MMPs	Degrades extracellular matrix, promotes plaque instability, and increases the risk of plaque rupture.	(91)

This table summarizes the major inflammatory mediators and regulatory molecules involved in the pathogenesis of atherosclerosis, together with their principal biological functions and representative references. These molecules collectively regulate endothelial dysfunction, inflammatory signaling, immune cell recruitment, oxidative stress, and plaque progression.

As lesions progress and plaque architecture becomes increasingly complex, the role of inflammatory mediators in regulating plaque stability becomes more pronounced. Persistent pro-inflammatory stimulation can modulate extracellular matrix metabolism by inducing upregulation of matrix metalloproteinase expression and suppressing collagen synthesis in vascular smooth muscle cells, thereby weakening the structural integrity of the fibrous cap. Concurrently, inflammatory cytokines promote apoptosis or necrosis of foam cells and other vascular wall cells, leading to progressive expansion of the necrotic core and a marked increase in the risk of plaque rupture and thrombosis (36). These processes indicate that inflammatory mediators not only drive plaque growth but also play a central role in determining the transition of plaques from a relatively stable state to an unstable phenotype.

Notably, atherosclerotic plaques do not contain exclusively pro-inflammatory signals; anti-inflammatory and repair-associated mediators are also involved in disease modulation. Anti-inflammatory cytokines represented by interleukin-10 (IL-10) and transforming growth factor-*β* (TGF-*β*) can, at specific stages, limit excessive inflammatory responses, promote tissue repair, and contribute to the maintenance of fibrous cap stability. The dynamic balance between pro- and anti-inflammatory mediators is therefore of critical importance for preserving plaque structural stability (37).

In addition, multiple inflammatory regulatory molecules continuously modulate the local inflammatory microenvironment of plaques by influencing endothelial homeostasis, immune cell adhesion and migration, lipid recognition, and oxidative stress responses. Rather than directly triggering inflammatory cascades, these molecules fine-tune disease progression by regulating the intensity and duration of inflammatory mediator activity (33, 38). Together with the classical inflammatory mediators described above, these regulatory molecules constitute an interconnected inflammatory network that governs plaque initiation, progression, and destabilization.

### 3.4 Inflammatory signaling pathways in atherosclerosis: focus on the NF-*κ*B and MAPK pathways

Inflammatory mediators within atherosclerotic plaques do not act in isolation but instead transduce extracellular stimuli into sustained inflammatory responses through intracellular signaling pathways. Within the chronic inflammatory microenvironment of atherosclerosis, pro-inflammatory cytokines, oxidatively modified lipids, and danger-associated molecular patterns collectively act on vascular wall–associated cells. By activating specific inflammatory signaling pathways, these stimuli are converted into persistent transcriptional responses and pathological alterations. Among the numerous inflammation-related signaling pathways that have been reported, the NF-*κ*B and MAPK pathways occupy central positions in integrating inflammatory mediator signals, maintaining chronic inflammatory states, and driving plaque progression, and they have been the most extensively investigated to date (39).

#### 3.4.1 The NF-*κ*B pathway

The NF-*κ*B pathway is one of the most classical inflammatory signal transduction pathways in atherosclerosis and can be activated in vascular endothelial cells, macrophages, and vascular smooth muscle cells by a variety of atherosclerosis-related stimuli. These include pro-inflammatory cytokines such as TNF-α and IL-1β, as well as oxidized low-density lipoprotein and pattern recognition receptor–mediated signals (40). Activation of NF-*κ*B induces the transcriptional expression of multiple inflammation-related genes, including cytokines, chemokines, and adhesion molecules, thereby amplifying local inflammatory responses at multiple levels.

In endothelial cells, NF-*κ*B–mediated expression of adhesion molecules and chemokines (such as VCAM-1 and MCP-1) enhances interactions between immune cells and the vascular wall, promoting the recruitment and retention of inflammatory cells at lesion sites (41). In macrophages, sustained activation of NF-*κ*B drives continuous release of pro-inflammatory cytokines, thereby maintaining a chronic inflammatory microenvironment within plaques and promoting foam cell–related pathological processes (42). These changes lead to persistent accumulation of inflammatory signals at the local level and prevent spontaneous resolution of inflammation. Based on these functional characteristics, the NF-*κ*B pathway is widely regarded as a key transcriptional regulatory hub linking inflammatory mediator networks with the pathological progression of atherosclerosis, and its sustained activation is closely associated with the long-term persistence of chronic inflammation in the vascular wall (43).

#### 3.4.2 The MAPK pathway

The MAPK pathway primarily participates in the amplification of inflammatory signaling and the regulation of cellular stress responses. This pathway consists of multiple functionally related branches and can respond to various pathological stimuli, including inflammatory signals, oxidative stress, and mechanical stress. In the atherosclerotic lesion environment, the MAPK pathway influences local inflammatory responses and plaque progression by regulating vascular wall cell proliferation, migration, apoptosis, and inflammatory cytokine expression (44).

In macrophages, activation of the MAPK pathway is closely associated with increased expression of pro-inflammatory genes and enhanced cellular stress responses, thereby promoting inflammatory amplification and sustained remodeling of plaque structure (45). In addition, specific MAPK branches participate in the regulation of cell fate–related processes such as proliferation and apoptosis, and alterations in their activity are correlated with necrotic core formation and increased plaque morphological complexity (46). Unlike the NF-*κ*B pathway, which primarily regulates inflammatory gene transcription, the MAPK pathway in atherosclerosis is more involved in sustaining inflammatory signal transmission and driving pathological progression.

#### 3.4.3 Coordinated regulation of the NF-*κ*B and MAPK pathways

Accumulating evidence indicates extensive functional crosstalk and coordinated regulation between the NF-*κ*B and MAPK pathways (47). In multiple vascular wall–associated cell types, both pathways can be simultaneously activated under shared inflammatory stimuli and mutually amplify each other at the signaling level, promoting sustained expression of inflammatory mediators. This cooperative regulation enhances the intensity of inflammatory responses and prolongs their duration. Such coordinated signaling enables the formation of a highly coupled regulatory network across different cell types and is considered a key molecular basis for the maintenance of chronic inflammatory states in atherosclerosis (48).

Sustained activation of the NF-*κ*B and MAPK pathways not only maintains local inflammatory responses but also further compromises plaque structural stability by affecting extracellular matrix metabolism, cell apoptosis, and oxidative stress levels, thereby creating a molecular environment favorable for plaque destabilization and subsequent thrombosis. Consequently, these two pathways are regarded as critical signaling hubs linking inflammatory mediator networks with the pathological outcomes of atherosclerosis and represent central targets of interest for current and potential therapeutic interventions. In addition to the NF-*κ*B and MAPK pathways, inflammatory regulation in atherosclerosis involves multiple other signaling pathways, the principal functions of which are summarized in Table 2.

**Table 2:** Inflammatory signaling pathways associated with atherosclerosis and their primary functions.

Signaling pathway	Primary effector cells	The primary function in atherosclerosis	References
NF-*κ*B	Endothelial cells, macrophages, smooth muscle cells	Induces the transcription of inflammatory mediators, chemokines, and adhesion molecules, promotes immune cell recruitment, sustains chronic inflammation, and drives plaque progression and destabilization.	(92)
MAPK	Macrophages, smooth muscle cells, endothelial cells	Amplify inflammatory signals, regulate cellular stress, proliferation, migration, and apoptosis, thereby promoting plaque structural remodeling and disease progression.	(93)
JAK/STAT	Immune cells, endothelial cells	Mediates cytokine signaling, enhances inflammatory responses and immune regulation, and participates in the amplification of inflammation.	(94)
PI3 K/Akt	Endothelial cells, smooth muscle cells	Regulating cell survival, metabolism, and functional states exerts bidirectional effects on endothelial function and plaque stability.	(95)
TGF-β/SMAD	Smooth muscle cells, fibroblasts	Promotes extracellular matrix deposition and fibrotic cap formation, thereby contributing to plaque stability maintenance to a certain extent.	(96)

This table summarizes major inflammatory signaling pathways involved in the pathogenesis of atherosclerosis, including NF-κB, MAPK, JAK/STAT, PI3 K/Akt, and TGF-β/SMAD pathways. The primary activating stimuli, principal effector cells, and core functions in disease progression are outlined. These pathways regulate inflammatory gene transcription, immune cell recruitment and activation, cellular proliferation and migration, oxidative stress responses, and extracellular matrix remodeling, thereby contributing to plaque formation, progression, and stability.

### 3.5 Mutual amplification of oxidative stress and inflammatory responses

Oxidative stress and inflammatory responses are two closely coupled pathological factors during the initiation and progression of atherosclerosis. Their persistent interaction within the vascular wall microenvironment constitutes an important basis for the difficulty in resolving chronic inflammation. Changes in reactive oxygen species (ROS) levels not only reflect the extent of cellular injury but also directly participate in the regulation of inflammatory responses, thereby preventing spontaneous resolution of local inflammation (49).

Elevated ROS levels can impair endothelial cell function and promote oxidative modification of lipid components such as low-density lipoprotein. Oxidized lipids activate endothelial cells and immune cells, inducing the expression of inflammatory cytokines and chemokines and thereby driving the local expansion of inflammatory responses.

As inflammatory reactions persist, activated immune cells further generate large amounts of ROS and related oxidative products, maintaining a high level of oxidative stress within the lesion microenvironment. Meanwhile, inflammatory mediators influence oxidative stress–related enzyme systems and intracellular redox balance, reducing the tolerance of vascular wall cells to oxidative damage and reinforcing the mutual amplification between oxidative stress and inflammation (49).

At the level of signaling regulation, ROS can modulate the activity of multiple inflammation-related signaling pathways, while sustained activation of inflammatory signaling further promotes ROS generation. This bidirectional interaction allows oxidative stress and inflammation to coexist over prolonged periods during plaque formation and progression and is closely associated with plaque structural disruption and alterations in plaque stability (50).

Recent studies have identified the NLR family pyrin domain containing 3 (NLRP3) inflammasome as a critical molecular link connecting oxidative stress with chronic vascular inflammation during atherosclerosis (51). Excessive ROS production promotes the dissociation of thioredoxin-interacting protein (TXNIP), which subsequently binds to and activates the NLRP3 inflammasome (52). Activation of the NLRP3 inflammasome triggers caspase-1 activation and promotes the maturation and release of the pro-inflammatory cytokines IL-1β and IL-18, thereby further amplifying local inflammatory responses. These cytokines, in turn, enhance oxidative stress and inflammatory signaling, forming a positive feedback loop that contributes to plaque progression and instability (53, 54). Therefore, the ROS–TXNIP–NLRP3 signaling axis is increasingly recognized as a key molecular mechanism underlying the reciprocal amplification between oxidative stress and inflammation in atherosclerosis.

### 3.6 Inflammation-Driven plaque destabilization and thrombosis

During the progression of atherosclerosis, persistent inflammatory responses not only promote plaque growth but also profoundly affect plaque structural stability, thereby serving as an important pathological basis for thrombosis. Under inflammatory conditions, plaques gradually transition from a relatively stable state to a vulnerable phenotype, rendering them more susceptible to rupture and erosion (55).

The sustained actions of inflammatory mediators and oxidative stress jointly weaken plaque structural integrity. On the one hand, inflammation-related signaling suppresses the reparative functions of vascular smooth muscle cells and promotes extracellular matrix degradation, leading to progressive thinning of the fibrous cap (56). On the other hand, apoptosis and necrosis of foam cells and other vascular wall cells result in expansion of the necrotic core, making plaques more prone to surface ulceration and rupture (57). Together, these changes substantially increase the risk of structural plaque disruption.

Meanwhile, inflammatory responses also alter the antithrombotic properties of the vascular endothelium. Following endothelial dysfunction, the vascular surface shifts from an anticoagulant and antiplatelet state toward a prothrombotic phenotype, creating favorable conditions for platelet adhesion and activation. In an inflammatory milieu, circulating platelets and the coagulation system exhibit heightened reactivity, rendering local vascular injury more likely to trigger thrombus formation (58).

In addition to platelet activation and coagulation abnormalities, increasing evidence indicates that neutrophil extracellular traps (NETs), generated through the process of NETosis, represent another important mechanism linking inflammation with thrombosis in atherosclerosis (59). NETs provide a prothrombotic scaffold that facilitates platelet adhesion, activation of coagulation factors, and fibrin deposition, thereby promoting thrombus formation following plaque rupture or erosion. In addition, NET-derived histones and proteolytic enzymes contribute to endothelial injury and amplify local inflammatory responses, establishing a vicious cycle between inflammation and thrombosis (59, 60). Consequently, NETosis has emerged as a critical contributor to plaque vulnerability and thrombotic complications in atherosclerosis.

Under the combined influence of these structural and functional abnormalities, plaque rupture or erosion can rapidly initiate platelet aggregation and thrombus formation, leading to acute or subacute occlusion of the vascular lumen. This process marks the transition of atherosclerosis from a chronic pathological condition to acute clinical events and represents a key mechanism underlying severe cardiovascular events such as myocardial infarction and ischemic stroke.

## 4 Mechanisms by which traditional Chinese medicine modulates inflammatory responses in atherosclerosis

**Figure 4:**
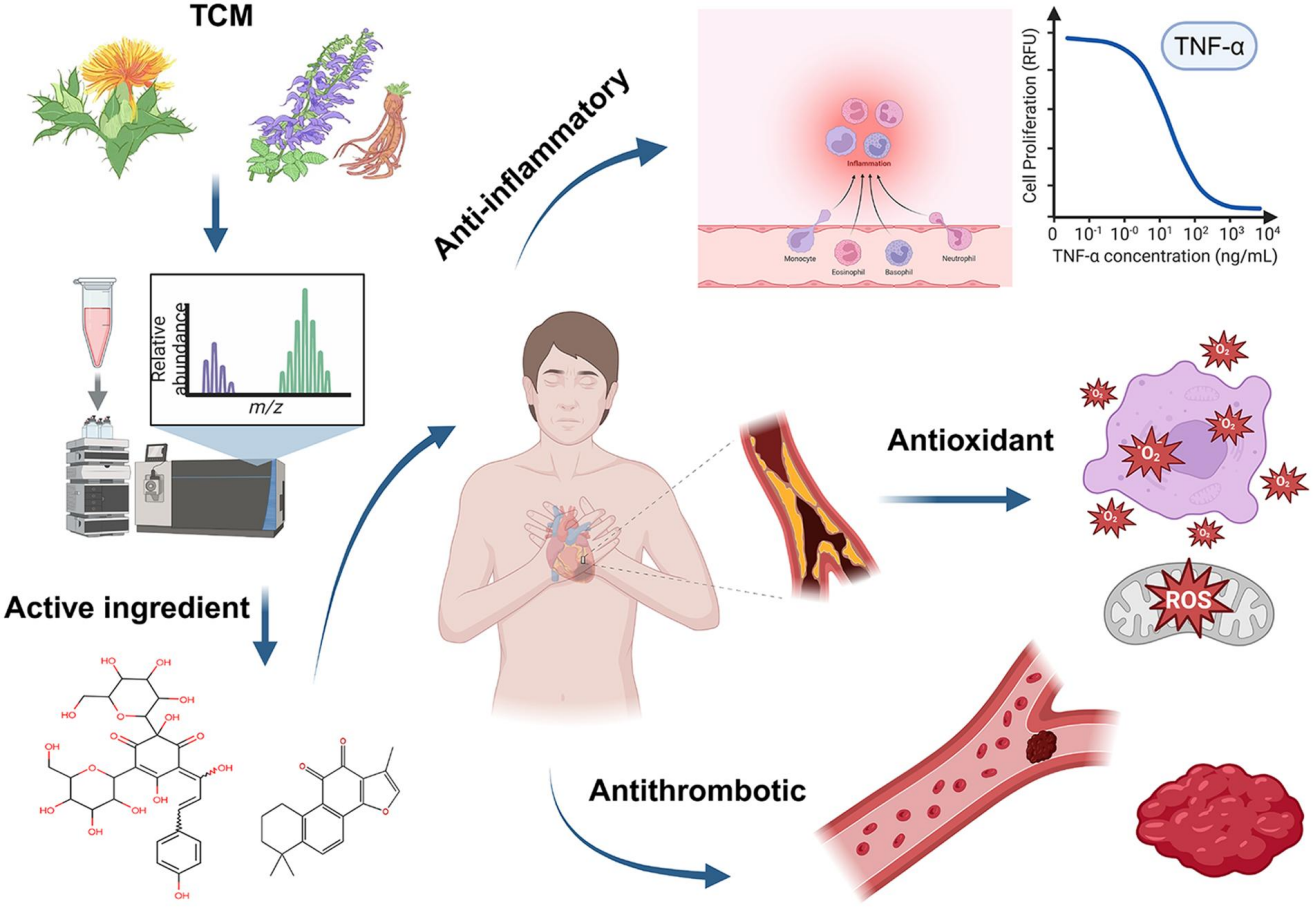
Mechanisms of traditional Chinese medicine in treatment this infographic illustrates the mechanisms of active ingredients from traditional Chinese medicine (TCM) in atherosclerosis, extracted using liquid chromatography-mass spectrometry. These active components exert anti-inflammatory effects by reducing levels of inflammatory mediators such as TNF-α, suppressing immune cell activity, and alleviating local inflammation; antioxidant effects by decreasing ROS production and reducing oxidative stress on the vascular endothelium; and antithrombotic effects by inhibiting platelet aggregation and lowering the risk of thrombus formation, thus slowing the progression of atherosclerosis.

Given that inflammatory responses in atherosclerosis involve multiple pathological dimensions, including metabolic dysregulation, endothelial dysfunction, immune imbalance, and thrombosis, single-target interventions often fail to achieve sustained and stable disease control. Against this background, therapeutic strategies based on TCM, characterized by multi-component, multi-target, and holistic regulatory properties, provide a novel research perspective for the prevention and treatment of inflammation-driven atherosclerosis (Figure 4). To provide a clearer overview of the anti-inflammatory actions of TCM in atherosclerosis, we systematically summarized representative traditional Chinese medicines and their underlying mechanisms in Table 3**.** These interventions target multiple inflammatory pathways, including metabolic regulation, endothelial protection, immune modulation, and inhibition of thrombotic signaling, thereby contributing to plaque stabilization and disease control.

**Table 3:** Traditional Chinese medicines (TCM) and their anti-inflammatory mechanisms in atherosclerosis.

TCM/Active component	Primary regulatory target	Key mechanisms	References
*Crataegus pinnatifida* (flavonoids, organic acids)	Lipid metabolism and oxidative stress	Modulates lipid metabolism and reduces oxidative stress.	(61)
*Alisma orientale* (triterpenoids)	Lipid metabolic pathways	Promotes LDL clearance and regulates lipid-related signaling pathways.	(62)
*Ligusticum chuanxiong* (ligustrazine)	miR-34a-5p/Sirt1 signaling	Restores Sirt1 signaling, promotes eNOS expression, inhibits NF-κB p65 acetylation, and improves coronary microcirculation.	(64)
*Coptis chinensis* (berberine)	Akt/IRF3 signaling	Suppresses pro-inflammatory signaling cascades.	(65)
*Scutellaria baicalensis* (baicalin)	MAPK/NF-κB signaling axis	Inhibits MAPK and NF-κB activation.	(66)
*Astragalus membranaceus* (Astragaloside IV)	NF-κB and MAPK pathways; immunometabolic regulation	Inhibits inflammatory signaling and attenuates vascular inflammation.	(67, 75)
*Panax notoginseng* (Ginsenoside Rb1)	Pro-inflammatory cytokine expression	Suppresses pro-inflammatory cytokines and restores apoptosis–autophagy balance.	(68)
Lindera aggregata (Isolinderalactone)	NF-κB signaling pathway	Suppresses pro-inflammatory cytokine expression.	(69)
*Carthamus tinctorius (*Hydroxysafflor yellow A*)*	PI3K-Akt/mTOR-NF-κB axis	Inhibits NF-κB activation and pro-inflammatory cytokine expression.	(70)
*Paeonia suffruticosa* (paeonol)	Caveolin-1/NF-κB signaling	Upregulates caveolin-1 and inhibits NF-κB activation.	(71)
*Gardenia jasminoides* (geniposide)	FOS/MAPK signaling	Suppresses MAPK-mediated transcriptional activation.	(72)
Quercetin	p38 MAPK pathway	Inhibits p38 phosphorylation and reduces p16 expression	(73)
*Salvia miltiorrhiza* (tanshinones, Tanshinone IIA)	NF-κB and MAPK pathways; endothelial protection	Anti-inflammatory and antioxidant effects; inhibits NF-κB and MAPK activation.	(74)
*Polygonum cuspidatum* (resveratrol)	ROS generation and NADPH oxidase	Reduces ROS production and oxidative stress amplification	(76)
*Schisandra* spp. (Schisanhenol)	Oxidative stress–related receptor signaling	Inhibits oxidative stress–mediated signaling activation	(77)
*Ginkgo biloba* (ginkgolide B)	PAF receptor signaling	Antagonizes PAF receptor and inhibits platelet activation	(78)

This table summarizes representative traditional Chinese medicines and their active components discussed in this review, highlighting their primary regulatory targets and mechanisms in atherosclerosis-related inflammation. These agents exert multi-component and multi-target effects across different pathological dimensions, including lipid metabolic imbalance, endothelial dysfunction, inflammatory signaling amplification, oxidative stress, and thrombotic susceptibility, thereby contributing to the modulation of plaque development and stability.

### 4.1 Systemic regulatory effects of traditional Chinese medicine on inflammatory responses

Inflammatory responses permeate the entire course of atherosclerosis, from disease initiation and progression to the development of complications, and serve as a key driving force in disease evolution. As a complex disorder involving intertwined abnormalities in lipid metabolism, persistent immune–inflammatory activation, and vascular dysfunction, therapeutic strategies for atherosclerosis often face limitations associated with single-target interventions, including restricted efficacy and insufficient long-term benefits. In contrast, TCM demonstrates distinct advantages in systemic regulation. Through a multi-component, multi-target, and multi-pathway mode of action, TCM exerts comprehensive regulatory effects on inflammation associated with atherosclerosis. A defining characteristic of TCM-based therapy lies in its ability to restore dynamic homeostasis of the internal environment by coordinately modulating multiple pathological processes, rather than merely suppressing a single inflammatory target. Specifically, the holistic regulation of inflammatory responses by TCM is reflected in its coordinated intervention across different pathological dimensions of atherosclerosis.

With respect to dysregulated lipid metabolism, an important upstream driver of inflammatory activation, multiple studies have shown that medicinal herbs such as *Crataegus pinnatifida* (hawthorn) and *Alisma orientale* can indirectly alleviate inflammatory burden by regulating lipid metabolism and oxidative stress. Hawthorn extracts are rich in flavonoids and organic acids, which are thought to collectively contribute to lipid metabolism regulation and antioxidant effects, thereby reducing lipid oxidation and the generation of pro-inflammatory mediators (61). Triterpenoid compounds derived from *Alisma orientale* have been reported to promote LDL clearance and modulate lipid metabolism–related signaling pathways, leading to improved lipid profiles and reduced abnormal lipid accumulation (62). These two herbs exhibit complementary effects in lipid regulation and oxidative stress attenuation, and by jointly improving the metabolic milieu, they provide a favorable foundation for subsequent inflammatory modulation.

At the level of vascular protection and functional improvement, *Salvia miltiorrhiza* (Danshen) and *Ligusticum chuanxiong* (Chuanxiong), a commonly used herbal pair in the prevention and treatment of cardiovascular diseases, are representative examples in the regulation of inflammation-associated vascular injury. Sodium tanshinone IIA sulfonate, a water-soluble tanshinone derivative from *Salvia miltiorrhiza*, has been shown to protect HUVECs against homocysteine-induced endothelial injury by restoring AKT/MAPK phosphorylation and upregulating NNMT together with the SIRT1/NRF2/HO-1 antioxidant pathway, thereby reducing intracellular ROS accumulation and mitochondrial dysfunction (63). Meanwhile, ligustrazine derived from *Ligusticum chuanxiong* protects coronary microvascular function by suppressing miR-34a-5p and restoring Sirt1 signaling, thereby promoting eNOS expression, inhibiting NF-*κ*B p65 acetylation, alleviating endothelial dysfunction and platelet activation, and ultimately improving coronary microcirculatory function (64). Acting from distinct yet complementary perspectives—endothelial protection and hemodynamic regulation—these two herbs participate in the modulation of vascular inflammatory responses and help interrupt the pathological transition from inflammation to thrombosis.

During stages characterized by pronounced inflammatory activation, TCM interventions place greater emphasis on suppressing excessively amplified pro-inflammatory signaling while re-establishing immune homeostasis. Evidence indicates that berberine, a major alkaloid component of *Coptis chinensis* (Huanglian), can attenuate inflammatory responses in vascular-related cells by modulating pro-inflammatory signaling pathways such as Akt/IRF3 (65). Similarly, baicalein derived from *Scutellaria baicalensis* (Huangqin) exerts anti-inflammatory effects by activating the AMPK/Mfn-2 axis and suppressing downstream phosphorylation of ERK1/2, p38, JNK, and NF-*κ*B (66). Astragaloside IV has been shown to attenuate atherosclerotic inflammation by inhibiting PI3 K/Akt/mTOR phosphorylation, while culture-based bacterial counting demonstrated concurrent alterations in intestinal bacterial composition, including increased *Bifidobacterium* and *Lactobacillus* and decreased *Enterococcus* and *Clostridium* (67). Such intervention strategies reflect the systemic regulatory philosophy of TCM, which aims to control pathological inflammatory activation while simultaneously promoting restoration of homeostasis through coordinated regulation of inflammation, metabolism, and endothelial function.

Although the systemic regulatory characteristics of TCM provide a promising framework for the management of atherosclerosis, the complex interactions among multiple bioactive constituents remain incompletely understood. In addition, standardized approaches for evaluating the synergistic effects of multi-component therapies are still lacking, highlighting the need for more integrated mechanistic studies.

### 4.2 Multi-target regulation of inflammatory cytokines by traditional Chinese medicine

Dynamic changes in inflammatory cytokines directly determine the intensity and persistence of inflammatory responses during the course of atherosclerosis. Unlike potent strategies that aim to suppress a single inflammatory cytokine, TCM interventions emphasize the modulation of overall cytokine expression patterns through multi-component and multi-target actions. The objective of such interventions is not to completely block inflammatory responses, but rather to limit excessive activation of pro-inflammatory signals while guiding inflammation from a persistently activated state toward a controllable resolution phase, thereby alleviating chronic inflammatory burden within the vascular wall.

At the level of pro-inflammatory cytokine regulation, several bioactive components derived from Chinese medicinal herbs have been reported to act on key steps involved in cytokine production and release. Ginsenoside Rb1, derived from *Panax notoginseng* (Sanqi), attenuates vascular inflammation by suppressing pro-inflammatory cytokines while restoring the balance between apoptosis and autophagy through regulation of Bcl-2/Bax, cleaved caspase-3, cleaved caspase-9, LC3-II, and Beclin-1 (68). Similarly, Isolinderalactone, a sesquiterpene lactone isolated from *Lindera aggregata* (Wuyao), has been shown to reduce the expression of pro-inflammatory cytokines, including TNF-α, IL-6, and IL-1β, through inhibition of IKK*β* phosphorylation, p65 phosphorylation, and NF-*κ*B p65 nuclear translocation in atherosclerosis models (69). Together, these findings indicate that TCM-derived bioactive compounds regulate inflammatory cytokine production through distinct upstream molecular mechanisms, thereby reshaping the inflammatory microenvironment rather than merely suppressing individual inflammatory mediators.

Despite accumulating evidence supporting the cytokine-regulating effects of TCM, several important challenges and controversies remain. Most available evidence is derived from *in vitro* experiments or *ApoE*^−/−^ mouse models, whereas high-quality clinical studies are still scarce. In addition, different TCM compounds often converge on common inflammatory cytokine networks despite acting through distinct upstream mechanisms, making it difficult to determine their specific molecular targets and relative therapeutic contributions. Furthermore, it remains controversial whether the anti-inflammatory effects of TCM mediated through cytokine regulation are sufficient to prevent plaque progression and cardiovascular events. Addressing this question will require more standardized mechanistic studies together with well-designed clinical trials.

### 4.3 Regulatory effects of traditional Chinese medicine on inflammatory signaling pathways

The chronic inflammatory state of atherosclerosis depends on sustained activation of multiple intracellular inflammation-related signaling pathways, among which the NF-*κ*B and MAPK pathways are regarded as central hubs for integrating inflammatory signals.

#### 4.3.1 Regulatory effects of traditional Chinese medicine on the NF-*κ*B signaling pathway

With respect to regulation of the NF-*κ*B signaling pathway, bioactive components derived from TCM exhibit stratified intervention capabilities targeting different activation levels of the pathway. Activation of NF-*κ*B typically depends on phosphorylation and degradation of inhibitor *κ*B (I*κ*B) mediated by the I*κ*B kinase (IKK) complex and is coordinately regulated by multiple upstream signaling axes. In atherosclerosis-related inflammatory responses, this pathway remains persistently activated.

Hydroxysafflor yellow A, a major constituent of *Carthamus tinctorius* (Honghua), has been reported to suppress activation of the PI3K–AKT/mTOR–NF-*κ*B signaling axis, thereby reducing nuclear translocation of NF-*κ*B and downregulating the expression of pro-inflammatory cytokines such as TNF-α and IL-6. This effect attenuates inflammatory activation of vascular-associated cells in atherosclerosis models (70).

Distinct from interventions that modulate upstream activation axes of NF-*κ*B, paeonol derived from *Paeonia suffruticosa* (Danpi) exhibits inhibitory effects by targeting key regulatory nodes within the NF-*κ*B pathway. Studies have demonstrated that paeonol suppresses NF-*κ*B activation by upregulating caveolin-1 expression, thereby reducing NF-*κ*B p65 activation and decreasing the expression of inflammation-related factors, including TNF-α, IL-6, and VCAM-1. As a result, endothelial inflammatory responses are alleviated in atherosclerosis models (71).

#### 4.3.2 Regulatory effects of traditional Chinese medicine on the MAPK signaling pathway

Regarding modulation of the MAPK signaling pathway, TCM-derived bioactive compounds participate in multi-level regulation of atherosclerosis-associated inflammatory responses by intervening in different inflammation-related branches. The MAPK pathway remains persistently activated during atherosclerosis progression and regulates the maintenance and amplification of inflammatory responses through transcription factor networks and cell fate determination.

Geniposide, a monomeric component isolated from *Gardenia jasminoides* (Zhizi), has been shown to inhibit aberrant activation of the FOS/MAPK signaling axis, leading to downregulation of inflammation-related genes such as *FOS*, *NR4A1*, and *IL1A*. This suppresses macrophage polarization toward a pro-inflammatory phenotype and reduces the release of inflammatory cytokines such as IL-1β, thereby attenuating intraplaque inflammation in atherosclerosis models (72).

Beyond transcriptional regulation, the p38 branch of the MAPK pathway is also persistently activated in plaque macrophages during atherosclerosis, where it promotes cellular senescence and the development of a senescence-associated secretory phenotype through upregulation of p16. Quercetin, a naturally occurring flavonoid widely present in various medicinal herbs and plants, has been shown to inhibit phosphorylation of p38 MAPK, thereby reducing p16 expression. This suppresses macrophage senescence and the release of inflammation-related mediators, alleviates inflammatory responses, and delays plaque progression in atherosclerosis models (73).

#### 4.3.3 Coordinated regulation of the NF-*κ*B and MAPK signaling pathways by traditional Chinese medicine

During chronic inflammation in atherosclerosis, the NF-*κ*B and MAPK signaling pathways jointly participate in the initiation, amplification, and persistence of inflammatory responses through complex signaling crosstalk. Inhibition of a single inflammatory signaling axis is often insufficient to sustainably block compensatory activation of inflammation, whereas simultaneous intervention in multiple inflammatory pathways may more effectively limit chronic inflammatory amplification.

Studies have demonstrated that certain TCM-derived bioactive components exert coordinated inhibitory effects on both NF-*κ*B and MAPK signaling pathways in atherosclerosis models. Tanshinone IIA, an active constituent of *Salvia miltiorrhiza* (Danshen), has been reported to suppress phosphorylation of ERK1/2, JNK, p38, and NF-*κ*B p65 in *LDLR*^⁻/⁻^ mice and macrophage inflammatory models, thereby attenuating inflammatory cytokine production (74). Consistently, Astragaloside IV, a major bioactive component of *Astragalus membranaceus* (Huangqi), inhibited phosphorylation of MAPK signaling components (ERK1/2, JNK, and p38) and NF-*κ*B p65, accompanied by reduced expression of inflammatory mediators including IL-6, iNOS, and VCAM-1, thereby improving plaque stability in atherosclerosis models (75).

Collectively, these findings indicate that coordinated modulation of NF-*κ*B and MAPK signaling through distinct molecular targets contributes to the anti-inflammatory effects of TCM in AS.

Although increasing evidence indicates that TCM modulates NF-*κ*B and MAPK signaling pathways, several important questions remain unresolved. Most current studies investigate individual signaling pathways in isolation, whereas the extensive crosstalk among inflammatory signaling networks in atherosclerosis remains insufficiently understood. Moreover, many TCM compounds simultaneously modulate multiple upstream and downstream signaling pathways, making it difficult to identify the key molecular mechanisms responsible for their therapeutic effects. Future studies integrating systems biology, network pharmacology, and experimental validation will be essential to clarify these complex signaling interactions and facilitate the clinical translation of TCM-based therapies.

### 4.4 Effects of traditional Chinese medicine on oxidative stress and inflammatory responses

Given the critical role of oxidative stress in inflammation-associated atherosclerosis, increasing attention has been directed toward TCM–based interventions targeting oxidative stress, which are regarded as an important entry point for suppressing inflammatory amplification and slowing disease progression.

In terms of reducing oxidative stress levels, bioactive components derived from TCM can exert synergistic effects through multiple regulatory layers. Among these, resveratrol and its glycosylated derivatives, major active constituents of *Polygonum cuspidatum* (Huzhang), are considered important modulators linking oxidative stress and inflammatory responses. Previous studies have demonstrated that these compounds not only reduce intracellular and tissue levels of reactive oxygen species (ROS) but also suppress NADPH oxidase activity, thereby limiting oxidative stress at its source. This effect is accompanied by a concurrent reduction in inflammatory cytokine levels, collectively improving the oxidative stress–inflammation milieu (76).

Beyond directly reducing ROS burden, TCM-derived components also play key regulatory roles in the amplification of oxidative stress–related signaling. Schisanhenol, a lignan compound isolated from *Schisandra* species, has been reported to markedly attenuate oxidative stress–associated injury in oxidized low-density lipoprotein (oxLDL)-induced endothelial damage models. Mechanistically, Schisanhenol suppresses LOX-1 expression, thereby reducing downstream p38 MAPK phosphorylation and NF-*κ*B p65 nuclear translocation, ultimately alleviating endothelial inflammatory activation and monocyte adhesion (77).

Overall, TCM-mediated interventions against oxidative stress involve regulation of ROS generation, oxidative stress–responsive signaling, and downstream inflammatory activation, thereby disrupting the reciprocal amplification between oxidative damage and inflammation. By organically coupling antioxidant effects with anti-inflammatory mechanisms, this systemic regulatory strategy helps weaken the reciprocal reinforcement between oxidative damage and inflammation, providing an intervention approach with both etiological control and vascular protective value for the prevention and treatment of atherosclerosis.

Current evidence suggests that modulation of oxidative stress is an important mechanism underlying the anti-inflammatory effects of TCM. Nevertheless, several important limitations should be acknowledged. Most current studies evaluate oxidative stress primarily by measuring ROS production or oxidative stress-related biomarkers, whereas standardized biomarkers linking oxidative stress modulation to plaque stabilization and clinical outcomes are still lacking. Furthermore, oxidative stress is closely intertwined with inflammation, endothelial dysfunction, and lipid metabolism, making it difficult to determine the relative contribution of oxidative stress modulation to the overall therapeutic efficacy of TCM. Future studies integrating multi-omics approaches with well-designed clinical studies will be essential to clarify the mechanistic and clinical significance of oxidative stress modulation in atherosclerosis.

### 4.5 Regulatory effects of traditional Chinese medicine on thrombotic susceptibility

Severe clinical events associated with atherosclerosis are often not determined solely by plaque burden but rather arise from a prothrombotic state formed under the long-term influence of inflammatory responses, oxidative stress, and endothelial dysfunction. In this process, abnormal platelet activation, a shift of the coagulation system toward a procoagulant state, and impairment of the antithrombotic endothelial phenotype collectively promote thrombus formation.

In terms of reducing thrombotic susceptibility, TCM acts through coordinated regulation of platelet function, coagulation processes, and endothelial homeostasis. Several bioactive components derived from TCM have been reported to inhibit abnormal platelet activation and aggregation, thereby lowering the risk of thrombus formation. Ginkgolide B, a characteristic diterpene lactone isolated from Ginkgo biloba, attenuates thrombotic susceptibility primarily by antagonizing the platelet-activating factor receptor (PAF-R), thereby suppressing platelet activation and aggregation. In addition, Ginkgolide B suppresses Syk and p38 MAPK phosphorylation, leading to reduced calcium efflux and diminished release of platelet factor 4 (PF4) and CD40 ligand (CD40L), thereby further limiting platelet aggregation and thrombus formation (78). These findings suggest that TCM-derived components can reduce thrombotic susceptibility by modulating platelet activation and endothelial–platelet interactions without relying on potent anticoagulant effects.

Interventions by TCM against thrombosis do not target a single coagulation factor but instead reduce overall thrombotic risk through coordinated anti-inflammatory, antioxidant, and antiplatelet actions at multiple levels. This regulatory paradigm—aimed at lowering thrombotic susceptibility rather than directly blocking the coagulation cascade—confers unique advantages to TCM in the long-term management of atherosclerosis-associated thrombotic complications. From a holistic pathological perspective, such strategies may reduce the risk of acute thrombotic events following plaque rupture and thus hold potential significance for preventing adverse cardiovascular events related to atherosclerosis.

Although preclinical studies have demonstrated promising antithrombotic effects of TCM, important gaps remain before these findings can be translated into clinical practice. Most current studies primarily evaluate platelet function, coagulation-related biomarkers, or thrombotic susceptibility in experimental models, whereas evidence demonstrating reductions in major adverse cardiovascular events remains limited. In addition, the balance between antithrombotic efficacy and bleeding risk has not been adequately investigated for many TCM-derived compounds. Future well-designed clinical studies are therefore needed to evaluate the long-term efficacy and safety of TCM-based antithrombotic strategies in patients with atherosclerosis.

## 5 Discussion and future perspectives

As an important pathological basis of cardiovascular diseases, atherosclerosis involves multiple interrelated pathological processes, including endothelial dysfunction, aberrant immune cell activation, chronic inflammatory responses, amplification of oxidative stress, and thrombosis (79). Among these processes, inflammation permeates the entire course of disease development, not only driving plaque formation and progression but also markedly increasing plaque instability and the risk of acute thrombotic events through its interactions with oxidative stress and the coagulation system (80). Given the central role of inflammation and its extensive crosstalk with other pathological processes, therapeutic strategies capable of simultaneously modulating multiple disease pathways may better match the complex pathophysiology of atherosclerosis. In this context, therapeutic strategies capable of simultaneously modulating multiple pathogenic pathways have attracted increasing attention, among which TCM represents a promising complementary approach.

Although modern medicine has developed a variety of therapeutic strategies targeting atherosclerosis, treatment paradigms centered on single targets still exhibit limitations in terms of long-term efficacy, safety, and system-level regulation (12). In particular, the CANTOS trial demonstrated that selective inhibition of IL-1β could reduce recurrent cardiovascular events, providing the first clinical evidence supporting inflammation as a therapeutic target in atherosclerosis. However, subsequent studies highlighted persistent residual inflammatory risk, patient heterogeneity, and concerns regarding long-term safety, indicating that single-target anti-inflammatory therapy alone may not fully address the complex pathophysiology of atherosclerosis (81). Furthermore, atherosclerosis is a multifactorial disease involving complex interactions among inflammation, oxidative stress, endothelial dysfunction, lipid metabolism, and thrombosis. Therefore, therapeutic approaches capable of simultaneously modulating multiple pathogenic pathways may better match the complex biological characteristics of the disease.

In this context, TCM, characterized by its multi-component, multi-target, and holistic regulatory properties, offers a research perspective distinct from conventional single-target strategies for intervening in inflammation-related pathological processes of atherosclerosis (13). Accumulating evidence indicates that TCM and its bioactive constituents can exert synergistic effects across multiple levels—including regulation of inflammatory cytokines, modulation of key signaling pathways, suppression of oxidative stress, and reduction of thrombotic susceptibility—thereby globally intervening in the core pathological network of atherosclerosis. From a mechanistic perspective, these therapeutic effects are achieved through coordinated regulation of multiple interconnected pathological processes, including inflammation, oxidative stress, endothelial dysfunction, and thrombosis, reflecting the systems-level mode of action of TCM (82). Nevertheless, the multi-target nature of TCM should also be considered critically. On the one hand, simultaneous regulation of multiple pathological pathways may better match the complex biological characteristics of atherosclerosis. On the other hand, the presence of numerous bioactive constituents with overlapping pharmacological activities complicates the identification of principal active components, mechanistic interpretation, and quality control, contributing to the perception of TCM as a mechanistic “black box”. Future studies integrating systems biology, network pharmacology, multi-omics analyses, and experimental validation will be essential to improve mechanistic understanding and facilitate clinical translation.

Despite the encouraging mechanistic findings from experimental studies, current clinical evidence supporting the use of TCM in atherosclerosis remains relatively limited. Several randomized controlled trials and meta-analyses have suggested that TCM formulations used as adjunctive therapy may improve inflammatory biomarkers, lipid metabolism, endothelial function, and certain surrogate indicators of atherosclerosis (83, 84). However, most available clinical studies are characterized by relatively small sample sizes, short follow-up periods, and considerable heterogeneity in study design, herbal formulations, and outcome measures. Moreover, robust evidence demonstrating reductions in major adverse cardiovascular events is still lacking. Therefore, large-scale, multicenter randomized controlled trials with standardized interventions and clinically relevant endpoints are required to further establish the efficacy and safety of TCM in the management of atherosclerosis.

Another issue deserving further attention is the stage-specific application of TCM during atherosclerosis progression. Early-stage atherosclerosis is primarily characterized by endothelial dysfunction and inflammatory activation, whereas advanced-stage disease is more closely associated with oxidative stress, plaque instability, and thrombotic complications. These pathological differences suggest that different TCM components may exhibit distinct therapeutic advantages at different stages of disease progression. For example, compounds targeting inflammatory cytokines and inflammatory signaling pathways may be more beneficial during the early inflammatory stage of atherosclerosis, whereas compounds with antioxidant and antithrombotic properties may play a greater role in maintaining plaque stability during advanced disease. However, current evidence remains insufficient to establish stage-specific therapeutic strategies. Future studies should clarify the optimal timing and combination of different TCM interventions, thereby facilitating the precision application of TCM in the management of atherosclerosis.

Looking forward, further integration of multi-omics analyses, systems biology, and precision pharmacology will be essential to elucidate the key regulatory nodes and synergistic mechanisms of Chinese herbal bioactive components within inflammatory signaling networks. Moreover, strengthening the linkage between basic research and high-quality clinical evidence will facilitate the standardized application and translational development of TCM in the prevention and treatment of atherosclerosis.
